# Male and female mice show significant differences in hepatic transcriptomic response to 2,3,7,8-tetrachlorodibenzo-*p*-dioxin

**DOI:** 10.1186/s12864-015-1840-6

**Published:** 2015-08-20

**Authors:** Jamie Lee, Stephenie D. Prokopec, John D. Watson, Ren X. Sun, Raimo Pohjanvirta, Paul C. Boutros

**Affiliations:** Informatics and Bio-computing Program, Ontario Institute for Cancer Research, Toronto, Canada; Department of Pharmacology & Toxicology, University of Toronto, Toronto, Canada; Department of Food Hygiene and Environmental Health, University of Helsinki, Helsinki, Finland; Laboratory of Toxicology, National Institute for Health and Welfare, Kuopio, Finland; Department of Medical Biophysics, University of Toronto, Toronto, Canada

**Keywords:** 2,3,7,8-tetrachlorodibenzo-p-dioxin, TCDD, Aryl hydrocarbon receptor, AHR, Sex differences

## Abstract

**Background:**

2,3,7,8–tetrachlorodibenzo-*p*-dixion (TCDD) is the most potent of the dioxin congeners, capable of causing a wide range of toxic effects across numerous animal models. Previous studies have demonstrated that males and females of the same species can display divergent sensitivity phenotypes to TCDD toxicities. Although it is now clear that most TCDD-induced toxic outcomes are mediated by the aryl hydrocarbon receptor (AHR), the mechanism of differential responses to TCDD exposure between sexes remains largely unknown. To investigate the differential sensitivities in male and female mice, we profiled the hepatic transcriptomic responses 4 days following exposure to various amounts of TCDD (125, 250, 500 or 1000 μg/kg) in adult male and female C57BL/6*Kuo* mice.

**Results:**

Several key findings were revealed by our study. 1) Hepatic transcriptomes varied significantly between the sexes at all doses examined. 2) The liver transcriptome of males was more dysregulated by TCDD than that of females. 3) The alteration of “AHR-core” genes was consistent in magnitude, regardless of sex. 4) A subset of genes demonstrated sex-dependent TCDD-induced transcriptional changes, including *Fmo3* and *Nr1i3*, which were significantly induced in livers of male mice only. In addition, a meta-analysis was performed to contrast transcriptomic profiles of various organisms and tissues following exposure to equitoxic doses of TCDD. Minimal overlap was observed in the differences between TCDD-sensitive or TCDD-resistant models.

**Conclusions:**

Sex-dependent sensitivities to TCDD exposure are associated with a set of sex-specific TCDD-responsive genes. In addition, complex interactions between the aryl hydrocarbon and sex hormone receptors may affect the observable differences in sensitivity phenotypes between the sexes. Further work is necessary to better understand the roles of those genes altered by TCDD in a sex-dependent manner, and their association with changes to sex hormones and receptors.

**Electronic supplementary material:**

The online version of this article (doi:10.1186/s12864-015-1840-6) contains supplementary material, which is available to authorized users.

## Background

Chlorinated dioxins are a large class of environmental contaminants generated as by-products of a variety of industrial processes [1]. Dioxin exposure can lead to a variety of toxic outcomes, and concerns surrounding widespread human exposure have led many of these to be extensively studied in model organisms [2–6]. To date, most studies of dioxin-induced toxicity have focused on the most potent and toxic congener, 2,3,7,8-tetrachlorodibenzo-*p*-dioxin (TCDD). At high doses, TCDD has been associated with numerous toxic outcomes in humans, including severe chloracne, neurotoxicity and tumourigenesis [3, 6–8]. In animal models, even small doses of TCDD have been shown to cause a wide range of toxicities, the severity and duration of which differ among species [2, 9, 10]. Although the exact mechanism of TCDD-induced toxicities is not completely understood, many studies have demonstrated that the interaction between TCDD and the aryl hydrocarbon receptor (AHR), a ligand-dependent transcription factor, plays a critical role in mediating them [11–13]. The AHR typically resides quiescently as a cytoplasmic complex with its chaperone proteins heat shock protein 90 (HSP90) and AHR-interacting protein (AIP). Upon ligand binding, the AHR translocates to the nucleus where it disaggregates from the chaperone proteins and. heterodimerizes with the AHR nuclear translocator (ARNT) [14]. The resulting complex binds to aryl hydrocarbon response elements (AHREs) in DNA and alters the transcription of target genes such as *Cyp1a1* [15–17].

The link between AHR-regulated transcriptional events and TCDD-induced toxicities was established by several experimental approaches. *Ahr* knockout mice demonstrated increased resistance to most TCDD-induced toxicities, relative to wild-type mice [9, 18]. Similarly, mice expressing mutations which prevent nuclear translocation, heterodimerization of AHR with ARNT, or AHRE binding are highly refractory to dioxin-induced toxicities [11, 19, 20]. In addition, mice lacking hepatic ARNT show reduced hepatotoxicity following treatment with TCDD [21]. These studies indicate that DNA binding of ligand-activated AHR is essential for the development of TCDD-induced toxic effects. In order to elucidate the specific mechanisms by which TCDD and the AHR elicit toxic outcomes, several groups have examined the AHR-mediated transcriptional events in various animal models and tissues following TCDD exposure [12, 13, 22–26]. Interestingly, the sensitivity of animals to TCDD-induced lethality was found to vary largely among species as well as between different strains within a species [27–30] (Table 1). One possible explanation for this variation involves the structure of the AHR: different isoforms were found to exist between species and strains which can be associated with the various toxicity phenotypes [31–33].

**Table 1:** TCDD sensitivity differences among animal models

Species	Strain	Male LD_50_ (μg/kg)	Female LD_50_ (μg/kg)
Hamster^a^	N/A	>5051	N/A
Guinea Pig^b^	N/A	0.6-2	N/A
Rat^c^	Han/Wistar (Kuopio)	>10000	>10000
	Long-Evans (Turku AB)	17.7	9.8
Mouse	C57BL/6*Kuo* ^d^	305	>5000
	DBA^e^	~2570	N/A

The dose-sensitivity of TCDD-induced lethality in animals varies largely among organisms; both inter- and intra-species variation exists

*N/A* Data not available

^a^Henck et al. [28]

^b^Schwetz et al. [27]

^c^Pohjanvirta et al. [30]

^d^Pohjanvirta et al. [36]

^e^Chapman and Schiller [29]

The role of the AHR becomes less clear when evaluating differences in toxic outcomes observed between male and female animals within the same strain [30, 34, 35]. While there was no difference in the sequence or structure of the AHR between male and female C57BL/6 mice, females were refractory to the typical toxic outcomes of TCDD [33, 36]. Other studies have demonstrated differential susceptibility of male and female C57BL/6 mice for a wide range of endpoints, including acute lethality, wasting syndrome, leukocytopenia and liver damage [36, 37]. Several studies suggest a complex interaction between the AHR and estrogen (ER) [38–41] and androgen receptors (AR) [39], which may partly explain the different TCDD-induced outcomes between sexes. However, the specific role of these receptors remains unclear. Female mice and guinea pigs are more resistant to TCDD-induced lethality than their male counterparts, while in rats, the sensitivity profiles are reversed, with females being the more TCDD-sensitive sex [30, 34, 36].

To further investigate this issue, a dose–response experiment was conducted on male and female C57BL/6*Kuo* mice and the hepatic transcriptomic profiles were evaluated with the intention of identifying sex-dependent/TCDD-mediated transcriptional events. Furthermore, a meta-analysis was performed using data from a similar time-course experiment in male and female mice [42] and using previous studies of rats under similar conditions [26, 43–45] in hopes of unravelling the mechanisms involved in producing the divergent TCDD-induced toxicities.

## Results

**Fig. 1:**
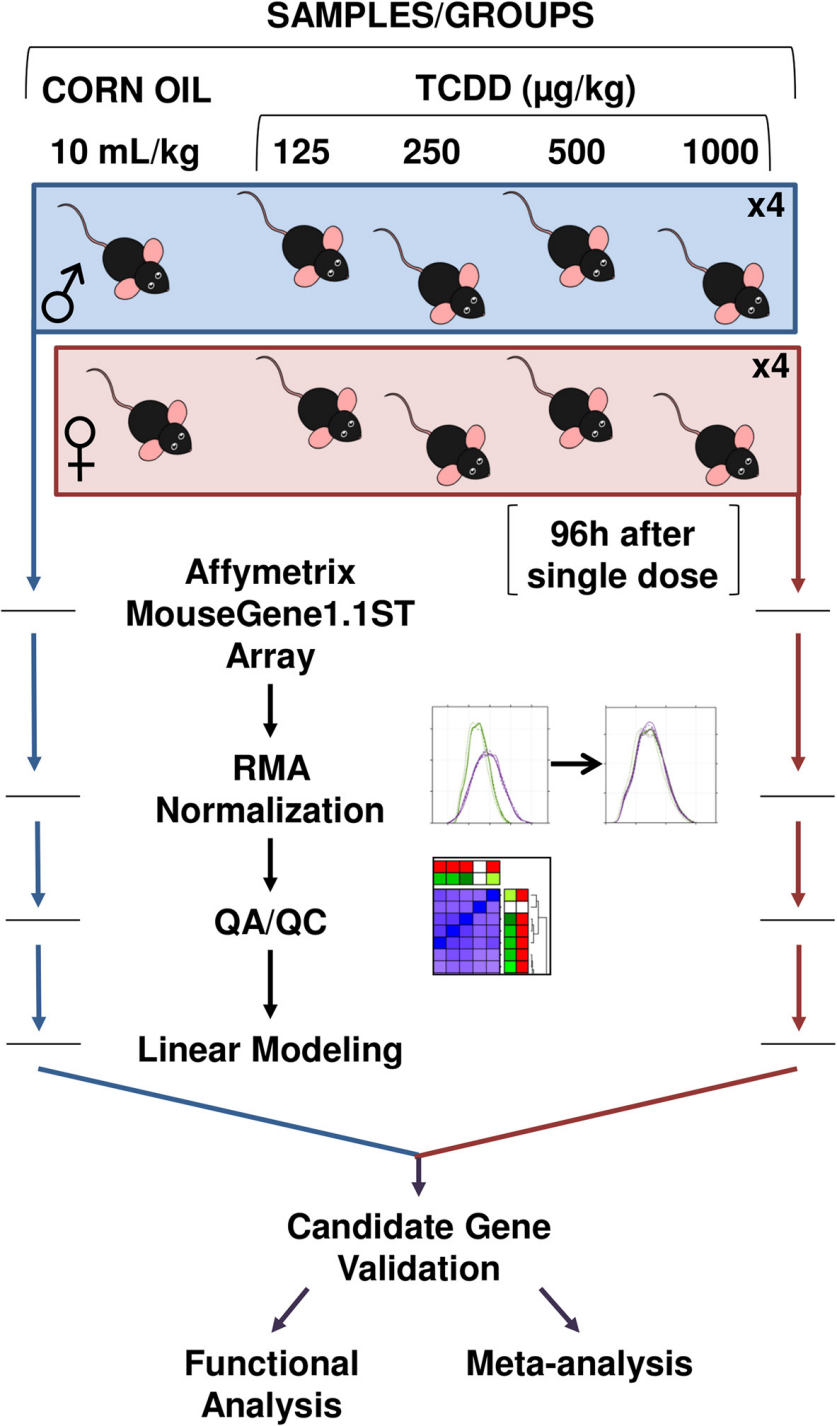
Experimental Design. Adult C57BL/6*Kuo* mice (20 each male and female) were divided into five treatment groups per sex. Each group received a single dose of TCDD in corn oil (125, 250, 500, or 1000 μg/kg) or corn oil alone. Livers were excised 4 days after treatment and RNA was isolated and hybridized to Affymetrix Mouse Gene 1.1 ST arrays. Data for each sex were pre-processed and modelled separately. Results for male and female cohorts were then combined. Those genes determined to be significantly altered by treatment were identified and downstream analyses, including pattern recognition and function analyses, were performed. A meta-analysis was performed through integration of data from 13 rodent-TCDD studies and additional analyses were performed to identify trends

To identify specific changes in mRNA abundance associated with differential TCDD-induced toxicities observed in male and female C57BL/6 mice, the hepatic transcriptome was profiled. Specifically, male and female C57BL/6*Kuo* mice were treated with either corn oil alone or a single dose of 125, 250, 500 or 1000 μg/kg TCDD in corn oil. It was previously demonstrated that survivor rates varied significantly between male and female animals within this range [36], with the lower TCDD doses being below the LD_50_ for male mice, and all doses being well tolerated by female mice (Table 1). In addition, a significant difference in plasma ALAT (alanine aminotransferase) activities between sexes existed 4 days post-exposure regardless of dose [36]. Therefore hepatic tissue was profiled 4 days after exposure to identify sex-specific transcriptomic changes (Fig. 1). It is important to note that while the higher doses of TCDD (500 and 1000 μg/kg) are lethal to male mice of this strain, lethality does not occur until day 14 at the earliest [36] hence animals were not yet moribund at this early time point.

### Overview of transcriptomic profiles

**Fig. 2:**
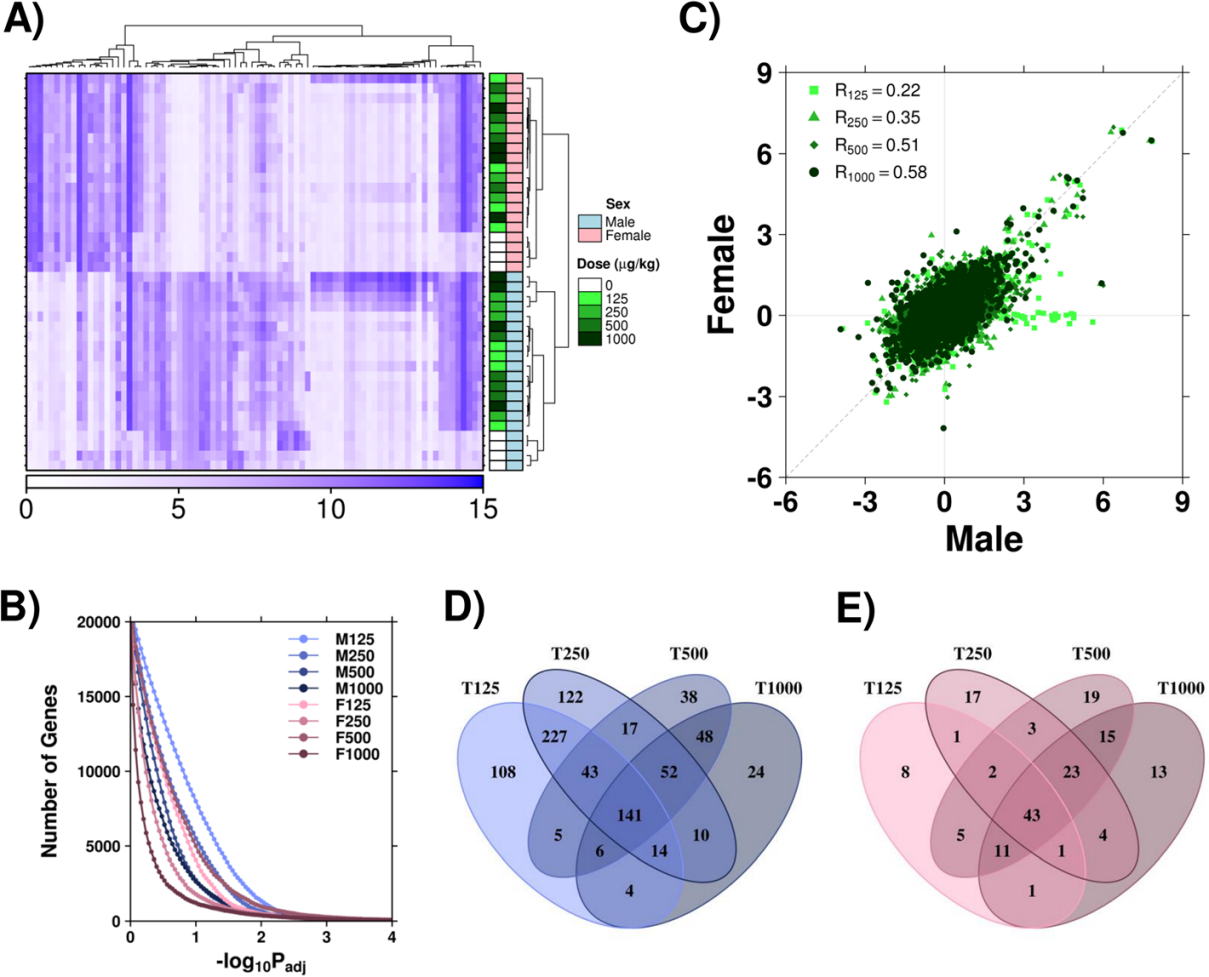
Summary of Transcriptomic Profiles. **a** RMA normalized intensity values for genes with the highest variance across all samples (variance > 2.0) were visualized; clear sex-specific and treatment-specific (TCDD or corn oil) abundance patterns were observed. Intensity values for each sample were clustered with the DIANA hierarchical clustering algorithm, with Pearson’s correlation as a similarity metric. Shading (white to blue) represents RMA normalized intensity values. **b** Linear modeling was performed to identify differences between treatment and control groups. Each sex was evaluated separately and results combined after modeling was applied. The legend indicates the experimental (*i.e*., M125 = male TCDD treated mice (125 μg/kg) relative to male vehicle control animals). The number of genes determined to be significantly altered at a range of FDR-adjusted *p*-value thresholds were examined across experimental groups. **c** Results (log_2_ fold-change) were compared between male and female mice for each dosage group. Pearson’s correlation indicated increasing similarity between transcriptomic profiles of male and female mice as TCDD dose was increased. Overlap of significantly altered genes following each dose of TCDD in (**d**) male and (**e**) female cohorts

Following data pre-processing, transcripts showing the most variable intensities were subjected to hierarchical clustering to visualize abundance patterns between treatment groups (Fig. 2a). Unsurprisingly, distinct transcriptomic profiles exist between male and female animals (independent of treatment), with further differences easily observed between TCDD treated and control animals within each sex. The Adjusted Rand Index (ARI) was calculated to quantitate this clustering. Cluster sizes of 2 (sex, treatment [treated or control]), 5 (dose [0, 125, 250, 500 or 1000 μg/kg TCDD]) and 10 (sex:dose) were evaluated. As expected, perfect agreement was identified for the partition based on sex (ARI = 1), moderate agreement existed for the combination of sex and dose (ARI = 0.26), while no agreement existed for treatment or dose alone (ARI = −0.02 and 0.03 respectively).

To quantify transcriptomic differences, linear modeling was performed to compare treated and control groups for each sex. Coefficients from the linear model provide the magnitude of difference between treated and control animals [log_2_(fold-change)], while modified *t*-tests were used to determine significance of differences between groups, followed by false discovery rate adjustment. The number of genes altered at various significance thresholds was assessed for each treatment group (Fig. 2b). In general, male mice showed more transcriptional changes following TCDD exposure than female mice at the same dose. To compare magnitude of change following treatment, the coefficients for each dose were compared between male and female mice, with Pearson’s correlation used to ascertain similarity across the transcriptome (Fig. 2c). The largest divergence between the sexes occurs at the lowest dose (125 μg/kg TCDD); a subset of transcripts showed increased abundance relative to control animals in only male animals following this dosage.

To further evaluate the extent of changes across the dose–response spectrum, the number of genes determined to be significantly altered (log_2_|fold-change| > 1 and *p*_*adj*_ *<* 0.01) were compared for each sex. Using these thresholds, male mice showed 141 transcripts altered across all doses, however a greater number of transcripts were consistently observed at doses below the LD_50_ for these animals (Fig. 2d). Conversely, females showed more genes altered at higher doses, and had only 43 transcripts altered at all four doses (Fig. 2e).

### Conserved transcriptomic responses

**Fig. 3:**
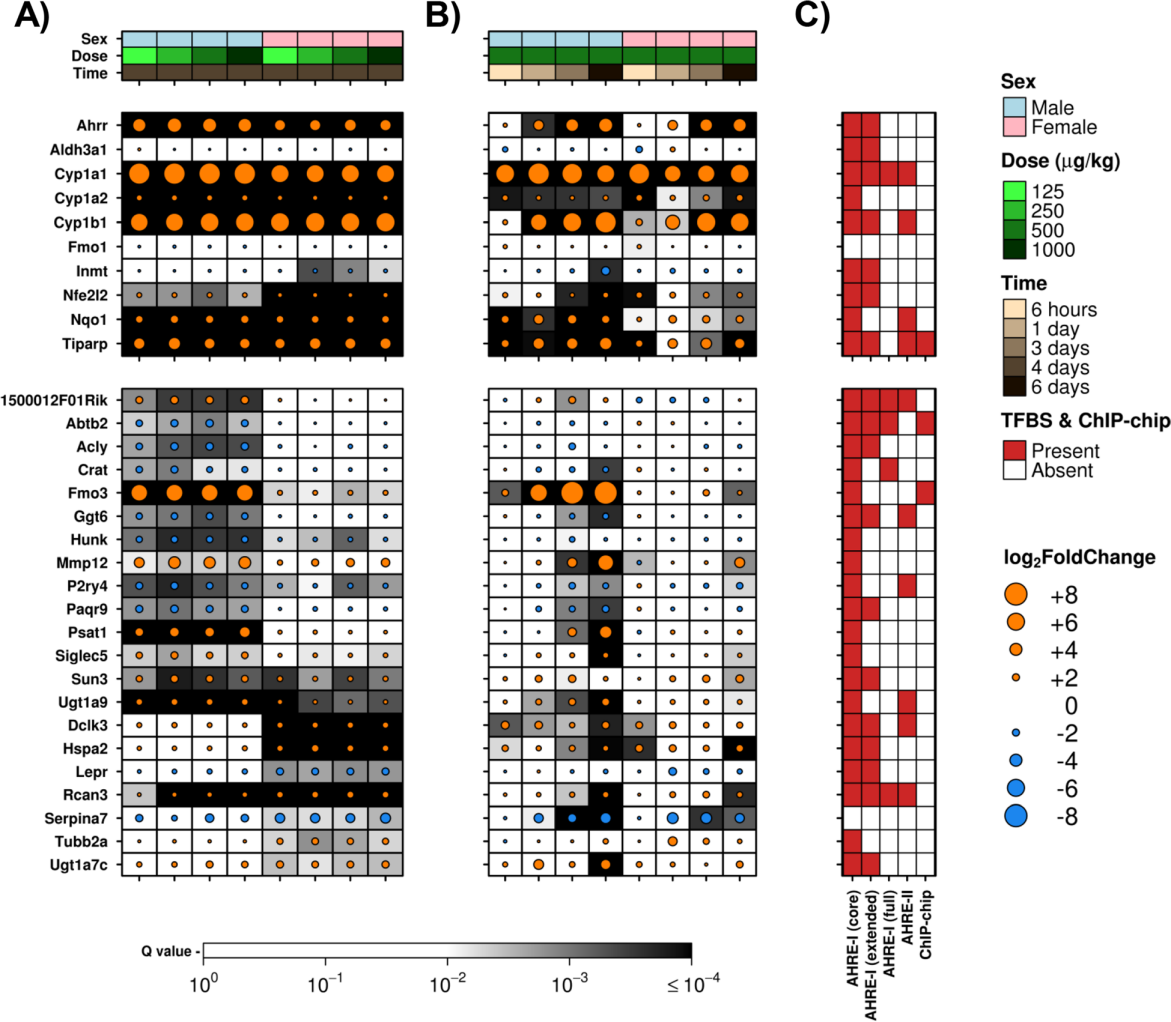
Transcriptomic Responses of Genes of Interest. Two subsets of genes were selected for visualization and comparison with additional datasets: 10 “AHR-core” genes and 21 genes determined to be significantly altered (|log_2_ fold-change| > 1.5 and *p*_adj_ < 0.01) following all four doses of TCDD in either male or female mice. The magnitude (dot size), direction (dot colour: orange and blue representing increased and decreased abundance respectively) and significance (background shading representing FDR-adjusted *p*-values) of changes relative to control animals are shown. Results for the “AHR-core” genes (top) and genes of interest (bottom) are shown from both (**a**) dose response and (**b**) time-course analyses. Covariates along the top indicate sample treatment while (**c**) demonstrate the presence or absence of AHRE motifs and AHR-binding, as determined by ChIP-chip analysis

Exposure to TCDD elicits transcriptional regulation through activation of the AHR [9, 11, 18, 19, 21]. Therefore, we examined 10 “AHR-core” genes (*i.e. Ahrr*, *Aldh3a1*, *Cyp1a1*, *Cyp1a2*, *Cyp1b1*, *Fmo1*, *Inmt*, *Nfe2l2*, *Nqo1* and *Tiparp*) that have previously been established to be altered following exposure to TCDD in various species and tissue types [12, 24, 46, 47]. These are genes typically involved in xenobiotic metabolism and the adaptive response to cellular stress. In general, the abundance profiles of these genes were similar between male and female mice (Fig. 3a, top dot-plot). Six genes (*Ahrr*, *Cyp1a1*, *Cyp1a2*, *Cyp1b1*, *Nqo1* and *Tiparp*) showed significant alteration in all cohorts. *Nfe2l2* demonstrated similar magnitudes of induction following TCDD exposure in all treatment groups; however differences in abundance between treated and control groups were more statistically significant in female mice. Similarly, *Inmt* was altered only in female mice (except at the lowest dose of TCDD, where it was unchanged); however the magnitude of induction was below our threshold. Therefore, none of the “AHR-core” genes were deemed to show sex-dependent differences in response to TCDD exposure.

The current dose response experiment only allows us to observe mRNA abundance changes 4 days after exposure. Therefore, results from a published time-course study [42] were added for comparison and the “AHR-core” genes were examined as above (Fig. 3b, top dot-plot). *Aldh3a1* and *Fmo1* were unaltered following TCDD treatment, regardless of dose or length of treatment. Interestingly, *Ahrr* and *Cyp1b1* were altered in livers of both male and female mice only at later time points. Differences in hepatic response between male and female mice for *Nqo1* and *Inmt* may be due to differences in the number of animals in each group between the studies (Table 2).

**Table 2:** Number of animals available per experimental group

Time (days)	Dose (μg/kg)	Number: Male (TCDD/Control)	Number: Female (TCDD/Control)
6 h^a^	500/0	5/4	5/4
1^a^	500/0	4/3	5/1
3^a^	500/0	4/4	4/1
4	125/0	4/4	4/4
4	250/0	4/4	4/4
4	500/0	4/4	4/4
4	1000/0	4/4	4/4
6^a^	500/0	4/3	5/2

The number of animals employed in each set of experimental conditions varied slightly between the current dose response and previous time-course analyses. At each time point, animals were treated with TCDD or vehicle control (indicated by Dose - TCDD/control). Numbers of male or female mice per group are shown as TCDD/control

^a^Prokopec et al. [42]

In order to better interpret the role of the AHR in regulating these genes, both transcription factor binding site (TFBS) analysis and AHR-binding analysis were performed (as described in methods). The presence or absence of various AHRE motifs, as well as the detection of AHR-binding by ChIP-chip for “AHR-core” genes is shown (Fig. 3c, top panel). Only *Fmo1* displayed an absence of AHRE motifs in the region examined, while only *Tiparp* demonstrated AHR-binding in this study.

### Sex-dependent transcriptomic responses

Since male and female C57BL/6 mice present divergent susceptibilities to dioxin-induced acute lethality, we sought to identify sex-dependent TCDD-responsive genes. Using the same dual threshold of log_2_|fold-change| > 1 and *p*_*adj*_ *<* 0.01 used above, 69 genes were significantly altered at all four doses in either male (37 genes) or female (32 genes) mouse liver. Overlap between male and female hepatic transcriptomic response following each TCDD dose is shown in Additional file 1. To further refine this list a more stringent threshold of log_2_|fold-change| > 1.5 and *p*_*adj*_ *<* 0.01 was applied, resulting in a set of 21 “candidate” genes with sex-dependent responses to TCDD (Fig. 3a, bottom dot-plot). Of these, the most notable sex-dependent response was observed for Flavin containing monooxygenase 3 (*Fmo3*). The protein product of this gene is involved in the oxidation of numerous xenobiotics. This gene is significantly induced following TCDD exposure in male livers, a result that has been described previously [48]. While *Fmo3* was significantly altered in all cohorts examined, the magnitude of change in female livers was smaller than that in male livers. Similarly, five additional genes were significantly altered across both males and females in the dose response study; however the magnitude of these changes reached the selected threshold in only male (*Hunk*, *P2ry4*, *Sun3* and *Ugt1a9*) or female liver (*Rcan3*).

The identification of sex-specific TCDD-responsive “candidate” genes was based solely on a single time-point and may be picking up secondary transcriptional events. However, the reduced TCDD sensitivity in female mice may be a result of rapid adaptive capabilities. Therefore, the “candidate” genes were further examined along time-course [42] (Fig. 3b, bottom dot-plot) to identify whether the candidates exhibit early or late changes. As expected, *Fmo3* mRNA abundance displayed an evident temporal response in male liver, with abundance increasing along the time-course. Many “candidate” genes (*i.e*., *Ggt6*, *Mmp12*, *Psat1*, *Rcan3* and *Serpina7*) showed altered abundance at only the later time-points examined (3 and/or 6 days post-exposure), likely indicating secondary responses to exposure. Strikingly, *Dclk3*, which encodes a protein kinase, displayed significantly altered hepatic abundance in only female mice along the dose response study, but was primarily altered in only male mice along the time-course study, with modest changes observed at 6 h post-exposure in females [42]. Also of interest, uncharacterized protein LOC68949 (*1500012F01Rik*, also known as *Zfas1*) – a lncRNA implicated in guiding site-specific methylation of rRNAs and moderately expressed in normal liver of male mice [49] – showed increased abundance in male hepatic tissue at all doses at 4 days post-exposure, as well as at 3 days post-exposure in the time-course study. In contrast, this transcript showed decreased abundance at early time-points in female hepatic tissue (though the change was not statistically significant).

As with the “AHR-core” genes, “candidate” genes were examined for AHRE motifs and AHR-binding (Fig. 3c, bottom panel). Although AHRE motifs were detected in most candidate genes (20/21), only two (*Abtb2* and *Fmo3*) demonstrated AHR-binding by ChIP-chip [50]. *Abtb2* was significantly down-regulated following treatment (all 4 doses at 4 days) in only male livers. No AHRE motifs were discovered within the searched region of the *Serpina7* gene, which was significantly down-regulated in female livers along the dose response, as well as in both male and female livers at later time points [42].

### Functional analysis TCDD-responsive genes

**Fig. 4:**
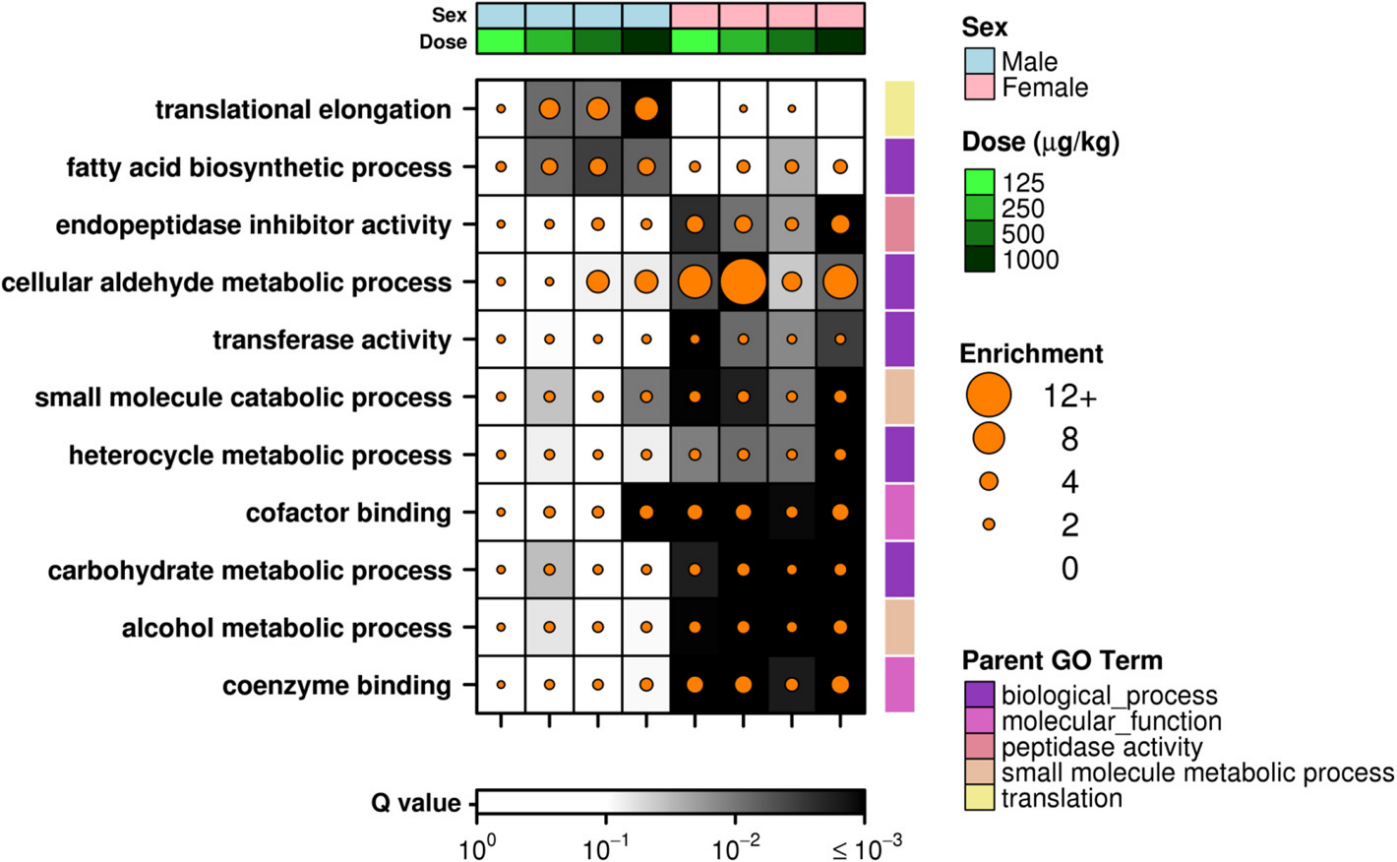
Pathway Analysis. Significantly altered genes (*p*_adj_ < 0.01) were identified for each sex/dose combination and used for pathway analysis (GOMiner). Gene ontologies that were determined to be significantly enriched (*p*_adj_ < 0.01) at multiple (3+) TCDD doses in either male or female mice are shown. Dot size indicates enrichment values while background shading represents FDR-adjusted *p*-values

To provide insight into the biological functions of TCDD-responsive genes in hepatic tissue of male and female mice, a pathway enrichment analysis was performed on the significantly altered genes (*p*_adj_ < 0.01) within each cohort. Gene ontologies that were significantly enriched (*p*_adj_ < 0.01) in each cohort were identified and comparisons were made between the sexes at each dose (Additional files 2 and 3). Surprisingly there were no significantly enriched GO terms in male liver at the lowest dose (125 μg/kg TCDD), despite this cohort having the most altered genes (Fig. 2b, Additional file 2A). Conversely, female liver showed 35 enriched terms at this low dose, despite a fewer number of genes. At higher doses, male and female hepatic tissues demonstrate similar numbers and significant overlap (hypergeometric test, *p* < 0.01) of enriched pathways (Additional file 2B–D). We identified 11 GO terms that displayed significant enrichment in a sex-specific manner (significant enrichment in 3+ cohorts in either sex). Interestingly, the majority of these were female-specific and include ontologies such as carbohydrate and alcohol metabolic processes (Fig. 4). Two pathways were enriched in a male-specific manner: translation elongation and fatty acid biosynthesis (Fig. 4). Interestingly, these pathways are enriched following only lethal doses [36].

### Alterations of TCDD-responsive genes in different biological contexts

**Fig. 5:**
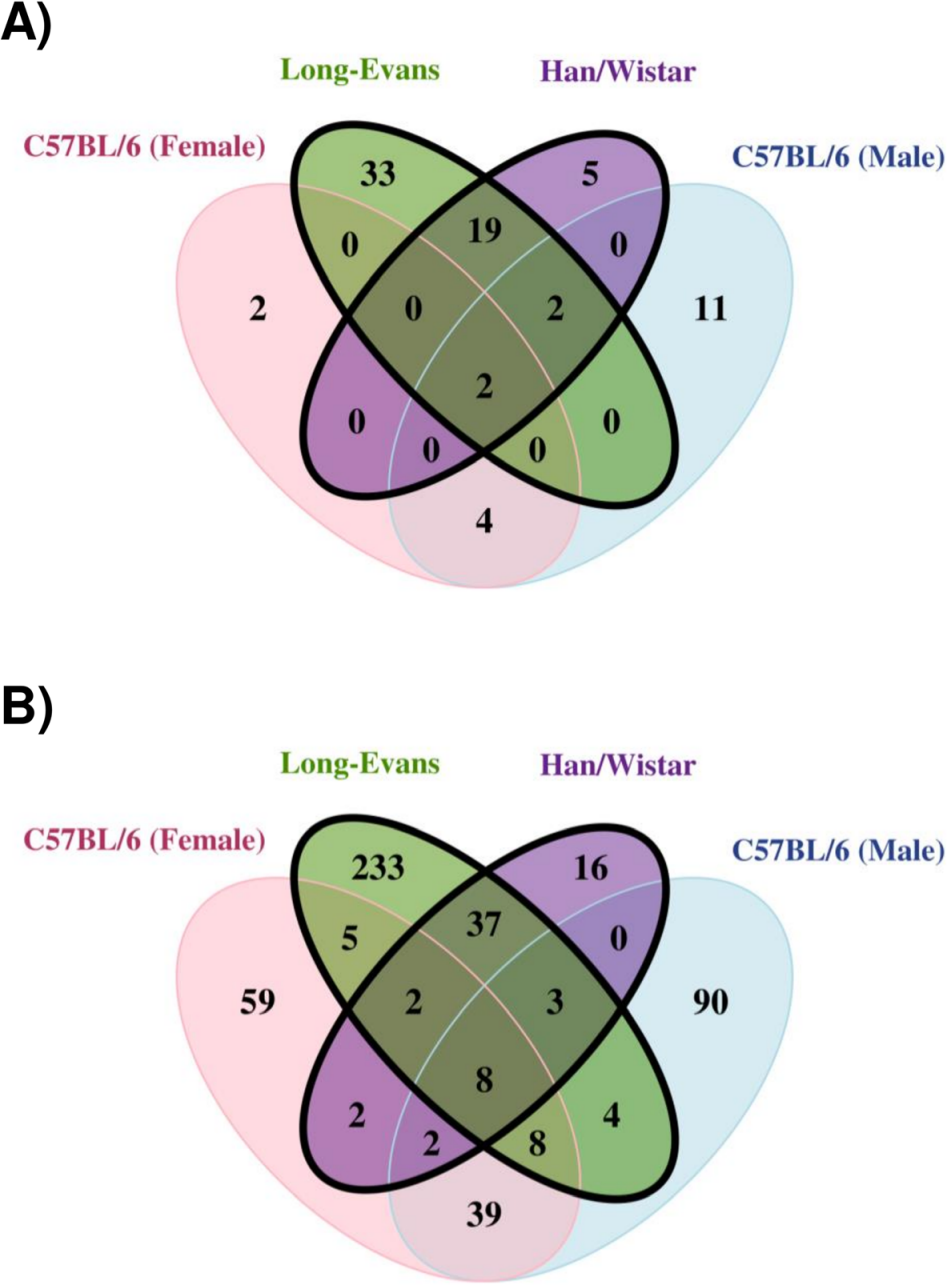
Meta-Analysis. The transcriptomic profiles for hepatic tissue from TCDD-treated mice and rats were compared. Mice were treated with 500 μg/kg TCDD while rats received 100 μg/kg – samples were collected at (**a**) 1 day and (**b**) 4 days after exposure. Only homologous genes were examined for consistency (*n* = 6871). Significantly altered transcripts were identified as those with log_2_|fold-change| > 1 and *p*_*adj*_ *<* 0.01

Finally, we sought to exploit our understanding of sex-associated transcriptional profiles to identify candidate drivers of dioxin toxicities. Because male and female mice show differential sensitivity to dioxin toxicities, we hypothesized that genes showing transcriptional differences between them might also drive differential sensitivity to dioxin toxicities in other model systems. We therefore integrated sex-specific changes with TCDD-dependent transcriptomic alterations in a variety of biological contexts (Table 3). These studies administered equitoxic doses of TCDD in sensitive rats and mice (100 and 500 μg/kg in rat and mouse respectively) [24]. Regardless of species, the transcriptomic changes induced by TCDD treatment were more pronounced in hepatic tissue, particularly at the later time-point examined. Few transcripts were statistically significantly altered in either hypothalamic or adipose tissues in rats. In hepatic tissue, a higher number of altered genes were observed in the dioxin-sensitive groups (C57BL/6 Male; Rat L-E Male) than in the dioxin-resistant groups (C57BL/6 Female; Rat H/W Male) both at 1 day (Fig. 5a) and 4 days (Fig. 5b) post-exposure. In hepatic tissue, there was minimal overlap among phenotypic groups, 4 days after treatment.

**Table 3:** Summary of datasets for meta-analysis

Species	Strain	Tissue	Sex	TCDD (μg/kg)	Time (days)
Mouse	C57BL/6*Kuo*	Liver	Male	500	1^a^
					4^b^
			Female		1^a^
					4^b^
Rat	Long-Evans	Liver	Male	100	1^c^
					4^d^
		Adipose			1^e^
		Hypothalamus			1^f^
	Han/Wistar	Liver	Male	100	1^c^
					4^d^
		Adipose			1^e^
		Hypothalamus			1^f^

To examine common and divergent transcriptomic alterations under different biological contexts, multiple microarray studies employing various rodent models (multiple species, strains, tissues, sexes and treatment time-points) were incorporated into a meta-analysis

^a^Prokopec et al. [42]

^b^Lee et al. (current)

^c^Yao et al. [44]

^d^Boutros et al. [43]

^e^Houlahan et al. [45]

^f^Houlahan et al. [26]

With the exception of *Cyp1a1* and *Nqo1*, minimal conservation was observed among the response of “AHR-core” genes to TCDD exposure across studies. *Nfe2l2* and *Tiparp* were altered in hepatic tissue of all organisms, while altered *Aldh3a1* was limited to rat liver (Additional file 4, top panel). Outside of the “AHR-core” genes, 14 genes were determined to be significantly altered (log_2_|fold-change| > 1 and *p*_*adj*_ *<* 0.01) in both TCDD-sensitive cohorts (Additional file 4, center panel) while 5 genes were altered primarily in the resistant subtypes (Additional file 4, lower panel).

Of those genes showing a sex-specific response 4 days after exposure to TCDD, 97 demonstrated significantly altered abundance in only male liver (log_2_|fold-change| > 1 and *p*_*adj*_ *<* 0.01) while 68 were altered in only female liver (Additional file 5). When compared with other biological contexts, *Gpd2* was altered in only the liver of male mice (both 1 and 4 day time points) while *Gls2* was additionally altered in liver tissue of L-E rats at both 1 and 4 days after exposure (Additional file 5, left panel). In female mouse liver, *Gstp1* and *Ugdh* were altered at both time points however were not altered in any of the additional cohorts examined. Alternatively, *Eml4* and *Exoc3* were additionally altered in hepatic tissue of both rat strains at both time points, while *Il1r1* was altered in only the resistant subtypes (Additional file 5, right panel). Ultimately, no genes were significantly altered exclusively to all of the sensitive- or resistant- phenotypes examined suggesting that these diverse models of dioxin sensitivity and resistance are driven by different genes. However, *Gls2* was altered in livers of both the TCDD-sensitive male mouse and L-E rat. *Gls2* encodes a glutaminase involved in reducing levels of reactive oxidative species (ROS) [51]. The reduced abundance of this transcript observed in TCDD-sensitive organisms may lead to increased intracellular ROS and subsequent toxicities. Livers of both female mice and H/W rat demonstrated significant induction of *Il1r1* (an interleukin 1 receptor) suggesting an enhanced immune response by these organisms.

## Discussion

Sensitivities to TCDD-mediated toxicities differ vastly across animal models. We have previously studied the hepatic transcriptomic responses to TCDD exposure in various strains of rats and mice, where various AHR isoforms have been shown to play a role in mediating differential sensitivity phenotypes [13, 43, 44]. Unfortunately, identifying the specific mechanisms by which differential toxicity occurs has proven difficult. Recently, studies have attempted to minimize the effect of genetic variation among different species and/or strains through the use of transgenic mice [33]. The current study attempts to minimize the impact of genetic variation by exploiting the differences in dioxin-sensitivities among male and female animals within the same genetic background. Therefore, characterization of the sex-specific TCDD-mediated transcriptomic changes may provide valuable information on the mechanisms of divergent TCDD-induced toxicities, independent of the AHR.

Our examination of hepatic transcriptomic profiles along both dose response and time-course experiments revealed several key findings. First, the general response patterns to TCDD exposure of male and female mice in terms of hepatic mRNA abundance are evidently different between the sexes [42]. Of the ~12,000 actively expressed hepatic genes in mice [52, 53], ~72 % have been described as being sexually dimorphic, with a fairly equal split between female- and male-biased genes [52]. Following treatment with TCDD, livers of female mice displayed a smaller number of altered transcripts relative to their corn-oil-treated counterparts than did male livers. This difference between male and female hepatic response is consistent along the dose response, however this was not the case early in the time-course [42], suggesting that this is a result of the longer exposure time. These results affirm that exposure to TCDD induces more transcriptomic alterations in the livers of male mice regardless of dose and as early as 1 day after exposure. This suggests a relationship between the quantity of transcriptomic changes and the increased sensitivity to lethality observed in male mice [36].

Second, the “AHR-core” gene response is relatively conserved across all cohorts. Transcriptomic changes can be categorized as either primary, mediated directly by dioxin-activated AHR, or secondary, those changes brought about by the onset of toxicity. The observed conservation of the “AHR-core” gene responses between the sexes, in particular the induction of classic AHR-regulated genes, such as *Cyp1a1, Cyp1a2* and *Cyp1b1*, suggests that AHR activity alone may not be the key factor determining the differential phenotypic response. Further evidence for this hypothesis comes from previous studies of *Cyp1a1*-null mice [37] in which susceptibilities to TCDD toxicities, including acute lethality, remained consistently different between the sexes. In addition, response patterns of these mRNA species have been shown to be conserved in both the TCDD-sensitive Long-Evans (L-E) and TCDD-resistant Han/Wistar (H/W) rats [13, 43, 47]. This further suggests that a group of genes outside of the “AHR-core” subset may be responsible for the difference in TCDD-induced toxicities.

Third, as alluded to above, a subset of “candidate” genes that exhibit sex-dependent response patterns was found to exist. Therefore, it is hypothesized that changes in abundance of these genes may result in either harmful (males) or beneficial (females) effects regarding TCDD toxicity. Further, altered abundance of these “candidate” genes may lead to downstream changes in pathway activities, and ultimately result in the observed toxicity outcomes.

In particular, our results demonstrate strong induction of *Fmo3* by TCDD at all doses examined in hepatic tissue of male mice. While basal expression of Fmo3 is considerably higher in female liver than in male liver, this induction results in mRNA levels that are similar to those observed in TCDD-treated female liver. *Fmo3* has been extensively studied in TCDD-treated mouse models [48]. The expression pattern of *Fmo3* varies largely between mouse and rat - this enzyme is constitutively expressed at higher levels in livers of female mice but virtually absent in livers of adult males while, in rats, abundance levels appear to be independent of sex [54]. It is also important to note that sex steroids have opposite effects on abundance of *Fmo3* between the two species. Testosterone has been shown to suppress expression and activity of FMO3 in mice, while female sex hormones had the opposite effect [55]. Conversely, testosterone has been shown to positively regulate the FMO gene family, while treatment with estradiol reduced FMO expression in rats [56]. Similarly, *Nr1i3*, which encodes the constitutive androstane receptor (CAR), was shown to be significantly upregulated following all doses (log_2_|fold-change| > 1.5 following the 3 highest doses) of TCDD and multiple time points (p_adj_ < 0.01) in male mice. *Nr1i3* can also be significantly suppressed by testosterone exposure [57]. TCDD-mediated sex steroid reduction has been well-demonstrated in previous studies, where exposure of TCDD significantly decreased testosterone, progesterone and estradiol in an AHR-dependent manner [58]. Reduced testosterone levels following TCDD-exposure may relate to the increased abundance of *Nr1i3* observed in our studies [42]. In addition, complex interactions have been described between the AHR and the estrogen (ER) and androgen (AR) receptors, in which ligand-activated AHR acts as both a transcriptional co-regulator for and promotor of degradation of the ERα and AR [39]. Taken together, this suggests that TCDD treatment alters the actions of sex hormones, either directly or indirectly through modulation of receptor activity, negatively regulating testosterone activities in male mice, thereby reducing the suppression of *Fmo3*. The opposite effect of testosterone on *Fmo3* in rats may also explain the reverse patterns of sex-dependent sensitivity to TCDD exposure. Multiple biological pathways were identified that displayed significant enrichment of altered genes following exposure to TCDD in a sex-dependent manner. While none of these were directly related to the above discussion of sex steroids and receptors, female livers showed an enrichment of altered genes involved in the inhibition of peptidase activity and cofactor binding, as well as various metabolic processes. This may allow female mice to better handle toxic metabolites and oxidative stress brought on by exposure to TCDD. The livers of male mice demonstrated enrichment of pathways related to translational elongation and fatty acid biosynthesis. Many of those genes demonstrating altered abundance associated with the translational elongation pathway are ribosomal components. A connection could be hypothesized between expression of these rRNAs and the increased abundance of the lncRNA *1500012F01Rik* (*Zfas1*). Enhanced synthesis of fatty acids may relate to the decreased body weight experienced by male mice following exposure to TCDD.

## Conclusions

The divergent responses to TCDD exposure in male and female C57BL/6 mice have been verified at the transcriptomic level. The primary responses directly regulated by the classical AHR-activation pathway are consistent, regardless of sex. Several sex-specific TCDD-responsive genes have been identified in hepatic tissue which may be associated with the differential sensitivities to TCDD induced toxicities. Moreover, different biological pathways demonstrated a significant enrichment of altered genes following TCDD exposure between the sexes. Previous studies have demonstrated a complex interaction between ligand-activated AHR and the activities of sex hormones and receptors [39, 58]. The current findings indicate altered abundance of specific genes may be involved in the differential phenotypic toxicities observed in male and female mice following exposure to TCDD. Further work is necessary to fully understand the specific mechanistic roles of sex hormones and associated receptors in these TCDD-induced toxicities.

## Methods

### Animal handling

Adult female and male C57BL/6*Kuo* wild-type mice were obtained from the National Public Health Institute, Division of Environmental Health, Kuopio, Finland. The current substrain (C57BL/6*Kuo*) originates from C57BL/6 J mice and was generated through multiple generations of inbreeding. Mice were employed at the age of 12–15 weeks to ensure both males and females had reached maturity. To prevent aggressive behaviour among animals, mice were each housed individually in Marolon cages with Altromin 1314 feed (Altromin Spezialfutter GmbH & Co. KG, Lage, Germany) and tap water available *ad libitum*. The temperature in the housing environment was maintained at 21 ± 1 °C, with a relative humidity of 50 ± 10 % and 12/12 h artificial light/dark cycle. The study protocols were approved by the Finnish National Animal Experiment Board (Eläinkoelautakunta, ELLA; permit code: ESLH-2008-07223/Ym-23).

### Experimental design

The experimental design is outlined in Fig. 1. Briefly, a total of 20 male and 20 female C57BL/6*Kuo* mice were used in this experiment. Animals were equally divided into 5 groups for each sex (*n* = 4) such that the age and weight range of the animals was consistent between groups. Each group received a single dose of TCDD (125, 250, 500 or 1000 μg/kg TCDD) or corn oil vehicle alone administrated by oral gavage (10 mL/kg) between 0900 and 1300 h. All mice were euthanized four days after treatment by carbon dioxide asphyxiation, immediately followed by cardiac exsanguination. Tissues were harvested, immediately frozen in liquid nitrogen and stored at −80 °C. Hepatic tissue was shipped on dry ice to the analytical laboratory for processing. All animal handling and reporting comply with ARRIVE guidelines [59]. Information regarding individual animal treatment is provided in Additional file 6.

### Microarray hybridization

Samples were prepared as described previously [60]. Briefly, tissue was ground to a fine powder in liquid nitrogen using a mortar and pestle, and rapidly homogenized using a Brinkmann Polytron (Polytron PT1600E with a PT-DA 1607 generator). Total RNA was extracted using an RNeasy Mini Kit following manufacturer’s instructions (Qiagen, Mississauga, Canada). Quantitation was performed using a Nanodrop UV spectrophotometer (Thermo Scientific, Mississauga, ON) and RNA integrity was verified by electrophoresis with RNA 6000 Nano kits on an Agilent 2100 Bioanalyzer (Agilent Technologies, Santa Clara, CA, USA). All samples demonstrated RNA integrity scores greater than 8.5 and were subsequently used in downstream analyses. RNA was transported to The Centre for Applied Genomics (TCAG) at The Hospital for Sick Children (Toronto, ON) and assayed on Affymetrix Mouse Gene 1.1 ST arrays using recommended protocols.

### Microarray pre-processing

Raw quantitated microarray data (CEL files) were obtained from TCAG. Data were imported into the R statistical environment (v3.1.2) using the affy package (v1.44.0) of the BioConductor library [61]. Male and female data were pre-processed separately using the RMA algorithm [62] to avoid masking sex-specific effects. Distributional homogeneity of arrays was assessed to detect outliers (Additional files 7 and 8); no arrays were excluded. An updated mapping of probes to Entrez Gene IDs was performed using the mogene11stmmentrezgcdf (v19.0.0) package [63]. Raw and pre-processed microarray data from this study are available in the Gene Expression Omnibus (GEO) repository under accession GSE61038. Visualizations were generated using the lattice (0.20-29) and latticeExtra (0.6-26) packages for R.

### Statistical analyses and visualization

General linear modeling was employed separately for each sex to identify transcripts altered by each dose of TCDD, relative to basal abundance level. Expression profiles were modeled as being a linear univariate combination of a basal effect and a TCDD-induced effect using a gene-wise linear model. Linear modeling was performed using the limma (v3.22.1) package for R. The standard error of each coefficient was adjusted with an empirical Bayes moderation of the standard error [64]. Model-based *t*-tests were used to assess whether each coefficient was significantly different from zero, followed by false-discovery rate (FDR) adjustment for multiple-testing [65]. Annotated results are provided in Additional files 9 and 10 (male and female respectively). Patterns of transcript abundance in male and female mice were visualized using DIANA hierarchical clustering with Pearson’s correlation similarity metric. For downstream analyses, a dual threshold of |log_2_ fold-change| > 1 and *p*_adj_ < 0.01 was used to define significantly altered transcripts, unless otherwise specified. Overlap of significantly altered genes between groups was visualized using the VennDiagram package (v1.6.11) for R [66].

### Pathway analysis

Gene ontology enrichment, performed using the High-Throughput GoMiner program (application build 454; database build 2011–01), was used to identify pathways significantly impacted by TCDD treatment [67]. For each treatment group, a list of significantly altered genes was identified as described above. Each list was compared against a randomly drawn sample from the database using 1000 randomizations, all mouse databases and look-up options, all gene ontology (GO) evidence codes and ontology classes (molecular function, biological process and cellular component), with a minimum of five genes per GO term and significance threshold of FDR-adjusted p-value < 0.1. Overlap of enriched GO terms between male and female mice was visualized using Venn diagrams (Additional file 2). GO terms were deemed differentially enriched between male and female mice if they were significantly enriched (p_adj_ < 0.01) at 3+ doses in either male or female cohorts.

### Transcription-Factor Binding Site (TFBS) analysis

A transcription-factor binding site analysis was performed to target motifs associated with AHR transcriptional regulation. The mouse reference genome was searched for given motif sequences occurring within ±3 kbp from the transcription start site of each gene. REFLINK and REFFLAT tables (build mm9) were downloaded from the UCSC genome browser [68] on July 15, 2014 to annotate transcription start sites. Four motifs were examined: AHRE-I (core), AHRE-I (extended), AHRE-I (full), and AHRE-II, with sequences GCGTG, TNGCGTG, [T|G]NGCGTG[A|C][G|C]A, and CATG{N6}C[T|A]TG respectively [69, 70]. For each available gene, the number of doses at which the gene was determined to be significantly altered by TCDD (*p*_*adj*_ < 0.01) in each sex and the number of occurrences and conservation score for each motif are provided in Additional file 11.

### Chromatin Immunoprecipitation (ChIP) - chip analysis

To verify if alterations in mRNA abundance were associated with AHR binding to regulatory motifs, a publicly available chromatin immunoprecipitation with DNA microarray (ChIP-chip) dataset (GSE11850) [50] was analyzed as described previously [42]. Briefly, raw data was downloaded for samples treated with DMSO or TCDD (GSM299306, GSM299307, GSM299310 and GSM299311) and normalized using RMA with the oligo package (v1.28.2) in R (v3.1.0). The binary probe map (NCBI build 35) provided by Affymetrix with mappings to mm7 was used to associate probes with specific genomic locations. Probes were then annotated with specific gene symbols by linking the genomic location to the nearest gene (±1 kbp from the TSS) using cisGenome [71] and REFFLAT tables (build mm7) downloaded on June 2, 2014 from UCSC genome browser [68]. Unannotated regions were removed and a Student’s *t*-test performed to detect statistically significant regions (*p*_adj_ < 0.05) in the R statistical environment (v.3.1.0). For genes with multiple probe mappings, a single probe with the lowest *p-*value was kept for downstream analyses.

### Meta-analysis TCDD toxicology studies in rodents

To extend our findings of differential transcriptomic changes induced by TCDD treatment in a variety of biological contexts, 12 datasets were incorporated into a meta-analysis of TCDD-responsive genes. Datasets are summarized in Table 3 and are available on the GEO repository: rat liver (1 day - GSE31411; 4 day - GSE13513), rat hypothalamus (GSE61039), rat adipose (GSE18301), mouse liver (1 day - GSE61037). For each dataset, TCDD-treated animals were compared with the corresponding corn oil-treated control group using linear modeling, as described above. Two groups of genes were identified as being associated with sensitive (log_2_|fold-change| > 1 and *p*_*adj*_ *<* 0.01 in livers of male mice and L-E rats at 4 days post-exposure) or resistant (log_2_|fold-change| > 1 and *p*_*adj*_ *<* 0.01 in livers of female mice and H/W rats at 4 days post-exposure) phenotypes.

### Availability of supporting data

Data for this study are available from the Gene Expression Omnibus (GEO) repository under series GSE61038. Supplementary material, including results from the transcription factor binding site and gene ontology enrichment analyses are available as additional files.

## Additional files

Additional file 1:
**Transcriptomic Overlap.** Venn diagrams display the number of significantly altered genes (log_2_|fold-change| > 1 and *p*_*adj*_ *<* 0.01) in male and/or female liver following treatment with a single dose of (A) 125, (B) 250, (C) 500 or (D) 1000 μg/kg TCDD. (PDF 139 kb) Additional file 2:
**Overlap of Enriched Pathways.** Pathway enrichment analysis was performed using GOMiner software. Analyses were performed for each sex/dose combination. Enriched gene ontologies were compared between male and female mice following treatment with (A) 125, (B) 250, (C) 500 and (D) 1000 μg/kg TCDD. (PDF 126 kb) Additional file 3:
**Pathway Analysis.** Pathway analysis was performed using GOMiner software to identify enriched gene ontologies within each sex/dose cohort. Enrichment scores and FDR-adjusted *p*-values are provided. (XLS 2546 kb) Additional file 4:
**Universally TCDD-Responsive Genes in Multiple Biological Contexts.** Datasets from multiple TCDD toxicity studies in rodents were integrated together to visualize transcriptomic alterations of (top) “AHR-core” genes and TCDD-responsive genes in different biological contexts. Two groups of genes were identified as being associated with (middle) sensitive (log_2_|fold-change| > 1 and *p*_*adj*_ *<* 0.01 in livers of male mice and L-E rats at 4 days post-exposure) or (bottom) resistant (log_2_|fold-change| > 1 and *p*_*adj*_ *<* 0.01 in livers of female mice and H/W rats at 4 days post-exposure) phenotypes. (PDF 175 kb) Additional file 5:
**TCDD-Responsive Genes in Male or Female Mice Examined in Multiple Biological Contexts.** Datasets from multiple TCDD toxicity studies in rodents were integrated together to visualize transcriptomic alterations of TCDD-responsive genes (log_2_|fold-change| > 1 and *p*_*adj*_ *<* 0.01) identified as altered in only (left) male or (right) female livers, 4 days post-exposure to 500 μg/kg TCDD. (PDF 425 kb) Additional file 6:
**Sample Information.** A total of 20 male and 20 female mice were included in this experiment. Each animal was treated with either corn oil or a single dose of TCDD (125, 250, 500 or 1000 μg/kg) dissolved in corn oil. Liver tissue was collected 4 days after treatment. (XLS 31 kb) Additional file 7:
**Array QA/QC (Male Cohort).** To verify data quality, the distributional homogeneity of arrays (A) pre- and (B) post-RMA processing was assessed. In addition, (C) RNA degradation was evaluated across probes for each array and (D) the inter-array correlation was examined to identify potential outliers; all arrays appeared highly similar and none were excluded from downstream analyses. (PDF 418 kb) Additional file 8:
**Array QA/QC (Female Cohort).** To verify data quality, the distributional homogeneity of arrays (A) pre- and (B) post- RMA processing was assessed. In addition, (C) RNA degradation was evaluated across probes for each array and (D) the inter-array correlation was examined to identify potential outliers; all arrays appeared highly similar and none were excluded from downstream analyses. (PDF 397 kb) Additional file 9:
**Annotated Results (Male cohort).** Linear modeling was performed to identify differentially abundant transcripts between TCDD-treated and control samples. A total of 21,115 transcripts were assessed in this study. Transcripts were annotated with Entrez Gene ID, gene Symbol and chromosome. Coefficients representing log_2_ fold-change and FDR-adjusted *p*-values are given. (XLSX 3725 kb) Additional file 10:
**Annotated Results (Female cohort).** Linear modeling was performed to identify differentially abundant transcripts between TCDD-treated and control samples. A total of 21,115 transcripts were assessed in this study. Transcripts were annotated with Entrez Gene ID, gene Symbol and chromosome. Coefficients representing log_2_ fold-change and FDR-adjusted *p*-values are given. (XLSX 3730 kb) Additional file 11:
**Transcription Factor Binding Site Analysis.** The count of occurrences and conservation scores for each of four AHRE motifs [AHRE-I (core), AHRE-I (extended), AHRE-I (full), and AHRE-II] were determined for each gene (within 3kbp up- and downstream of the transcription start site). For comparison, the number of doses at which each gene was altered by TCDD (*p*_*adj*_ < 0.01) in either male or female mice is shown. (XLS 3783 kb)

## Abbreviations

TCDD: 2,3,7,8–tetrachlorodibenzo-*p*-dixion
AHR: Aryl hydrocarbon receptor
ARNT: Aryl hydrocarbon receptor nuclear translocator
AHRE: Aryl hydrocarbon response element
ARI: Adjusted Rand Index
TFBS: Transcription factor binding site
GO: Gene ontology
L-E: Long-Evans rat
H/W: Han/Wistar rat
