# Supplementary figures and images for: Male and female mice show significant differences in hepatic transcriptomic response to 2,3,7,8-tetrachlorodibenzo-p-dioxin
Source: BMC Genomics. 2015 Aug 20;16(1):625. doi: 10.1186/s12864-015-1840-6 (PMC4546048; doi:10.1186/s12864-015-1840-6)

**A)**

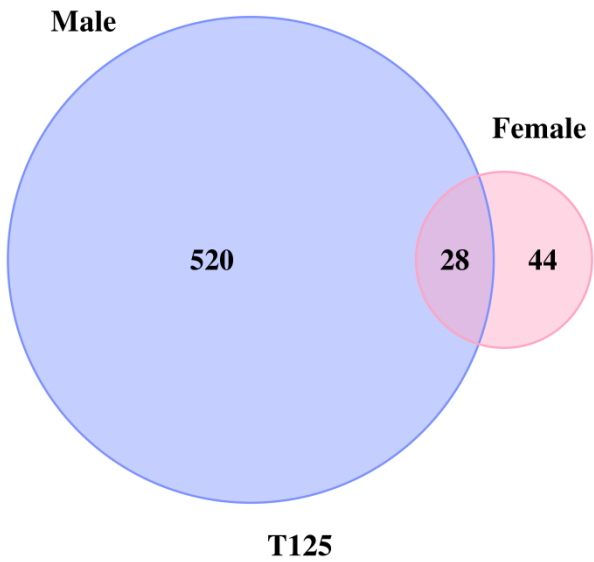

**B)**

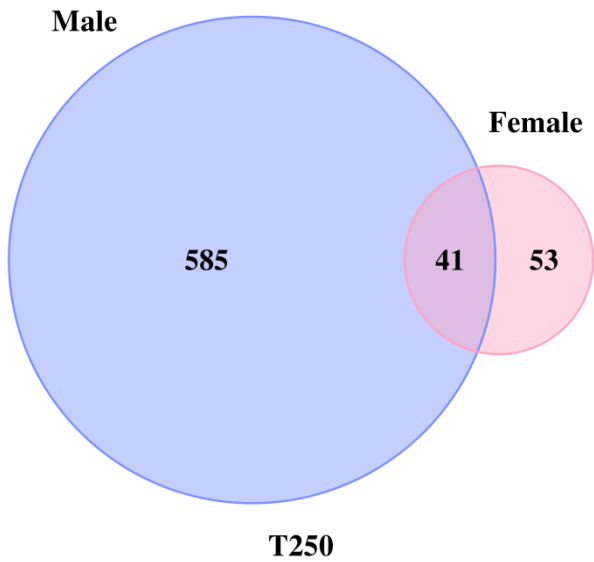

**C)**

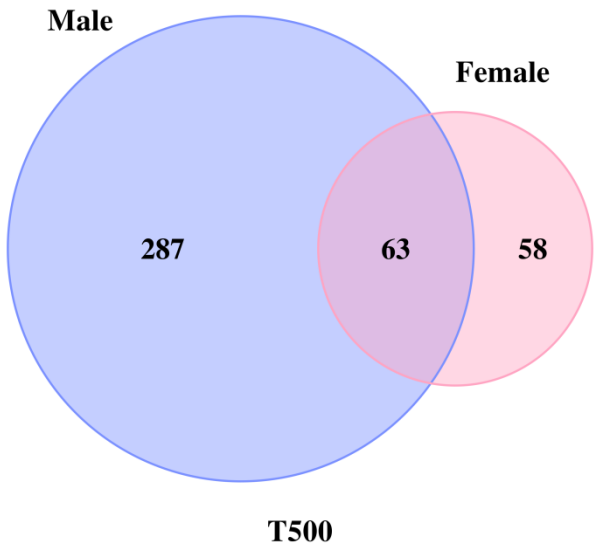

**D)**

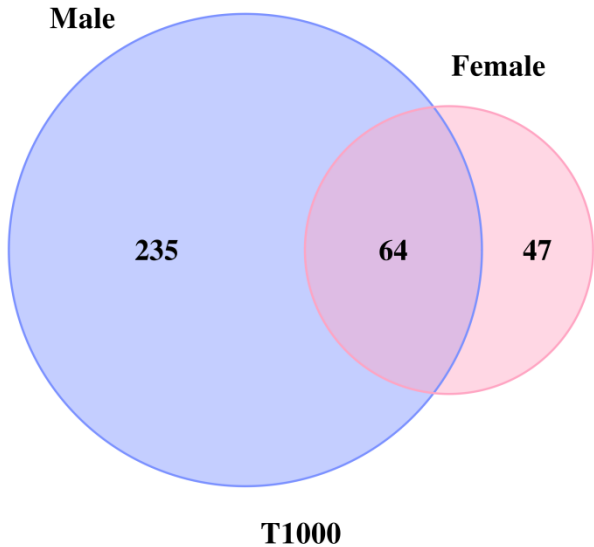

Supplement: Additional file 1: — Transcriptomic Overlap. Venn diagrams display the number of significantly altered genes (log2|fold-change| > 1 and p adj < 0.01) in male and/or female liver following treatment with a single dose of (A) 125, (B) 250, (C) 500 or (D) 1000 μg/kg TCDD. (PDF 139 kb) [file 12864_2015_1840_MOESM1_ESM.pdf]

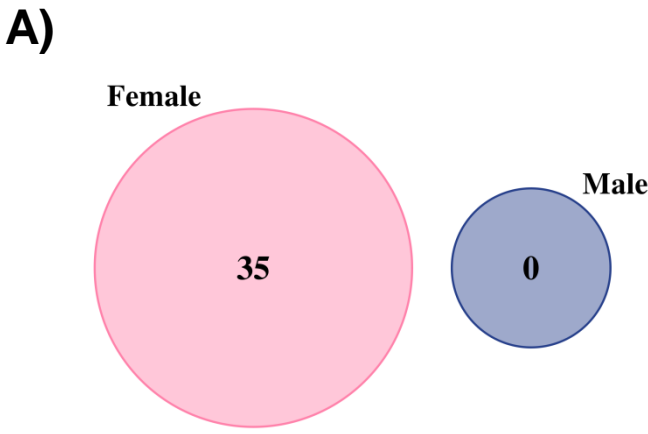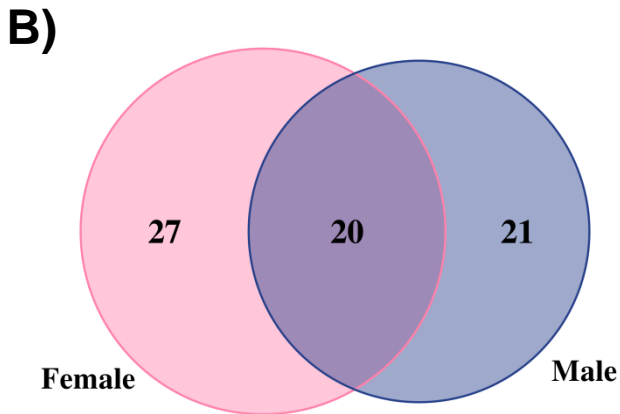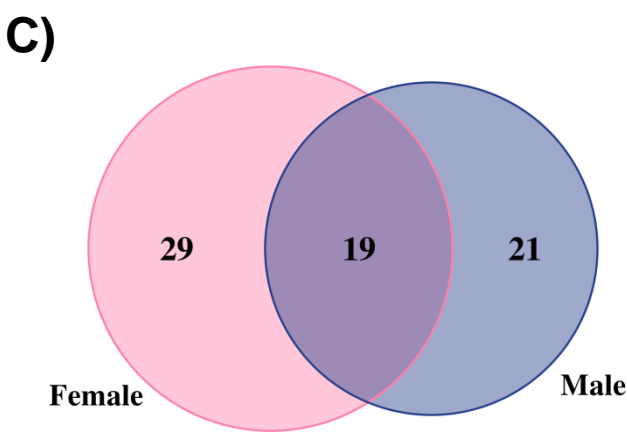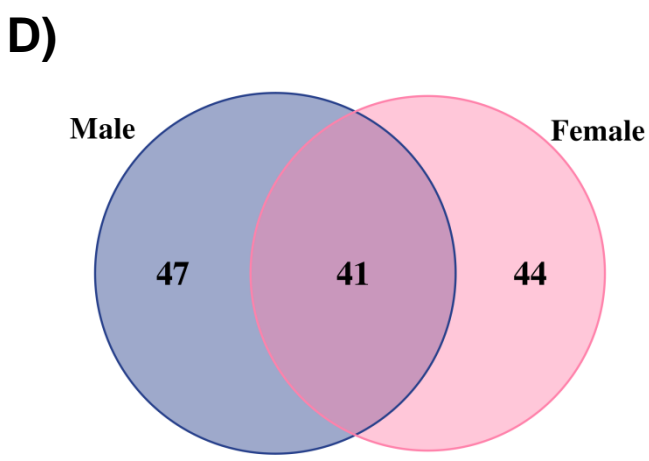

Supplement: Additional file 2: — Overlap of Enriched Pathways. Pathway enrichment analysis was performed using GOMiner software. Analyses were performed for each sex/dose combination. Enriched gene ontologies were compared between male and female mice following treatment with (A) 125, (B) 250, (C) 500 and (D) 1000 μg/kg TCDD. (PDF 126 kb) [file 12864_2015_1840_MOESM2_ESM.pdf]

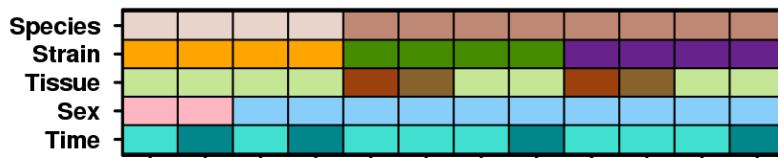

## Species

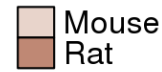

## Strain

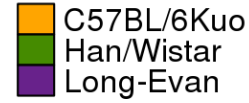

## Tissue

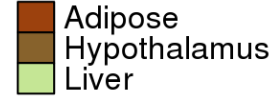

## Time

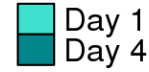

## Sex

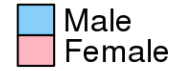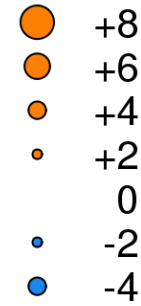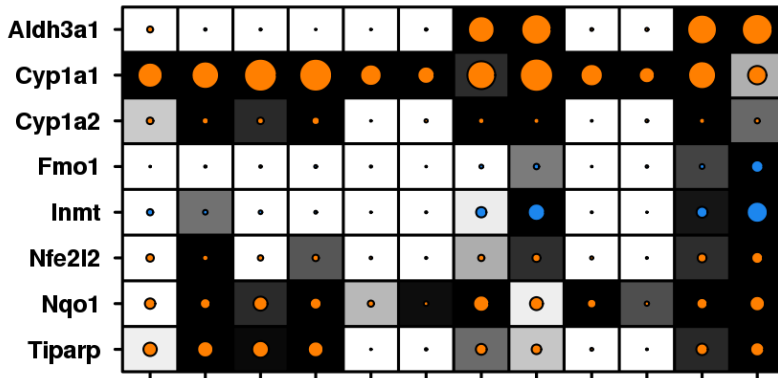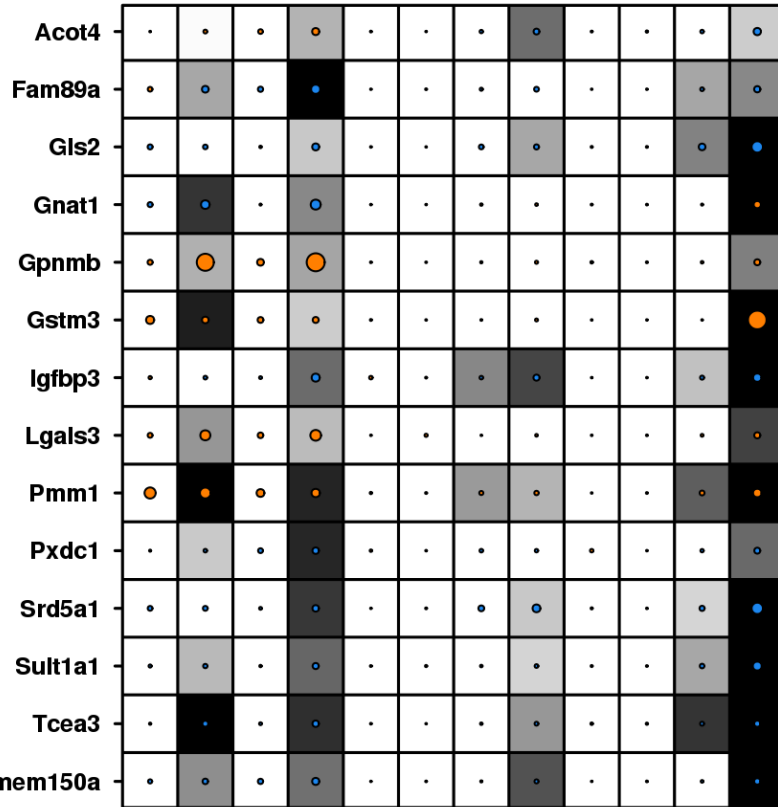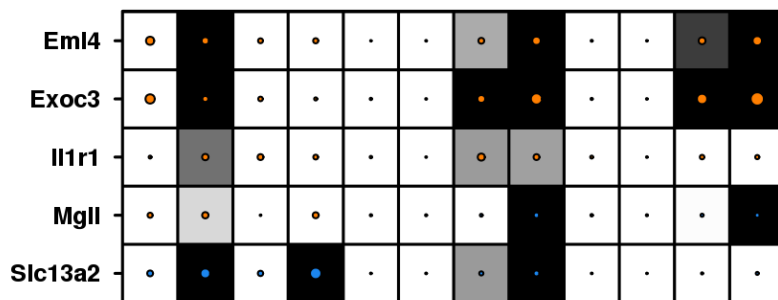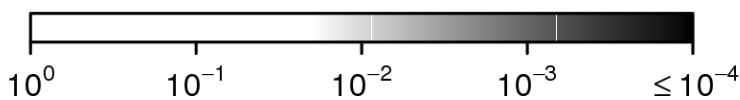

Supplement: Additional file 4: — Universally TCDD-Responsive Genes in Multiple Biological Contexts. Datasets from multiple TCDD toxicity studies in rodents were integrated together to visualize transcriptomic alterations of (top) “AHR-core” genes and TCDD-responsive genes in different biological contexts. Two groups of genes were identified as being associated with (middle) sensitive (log2|fold-change| > 1 and p adj < 0.01 in livers of male mice and L-E rats at 4 days post-exposure) or (bottom) resistant (log2|fold-change| > 1 and p adj < 0.01 in livers of female mice and H/W rats at 4 days post-exposure) phenotypes. (PDF 175 kb) [file 12864_2015_1840_MOESM4_ESM.pdf]

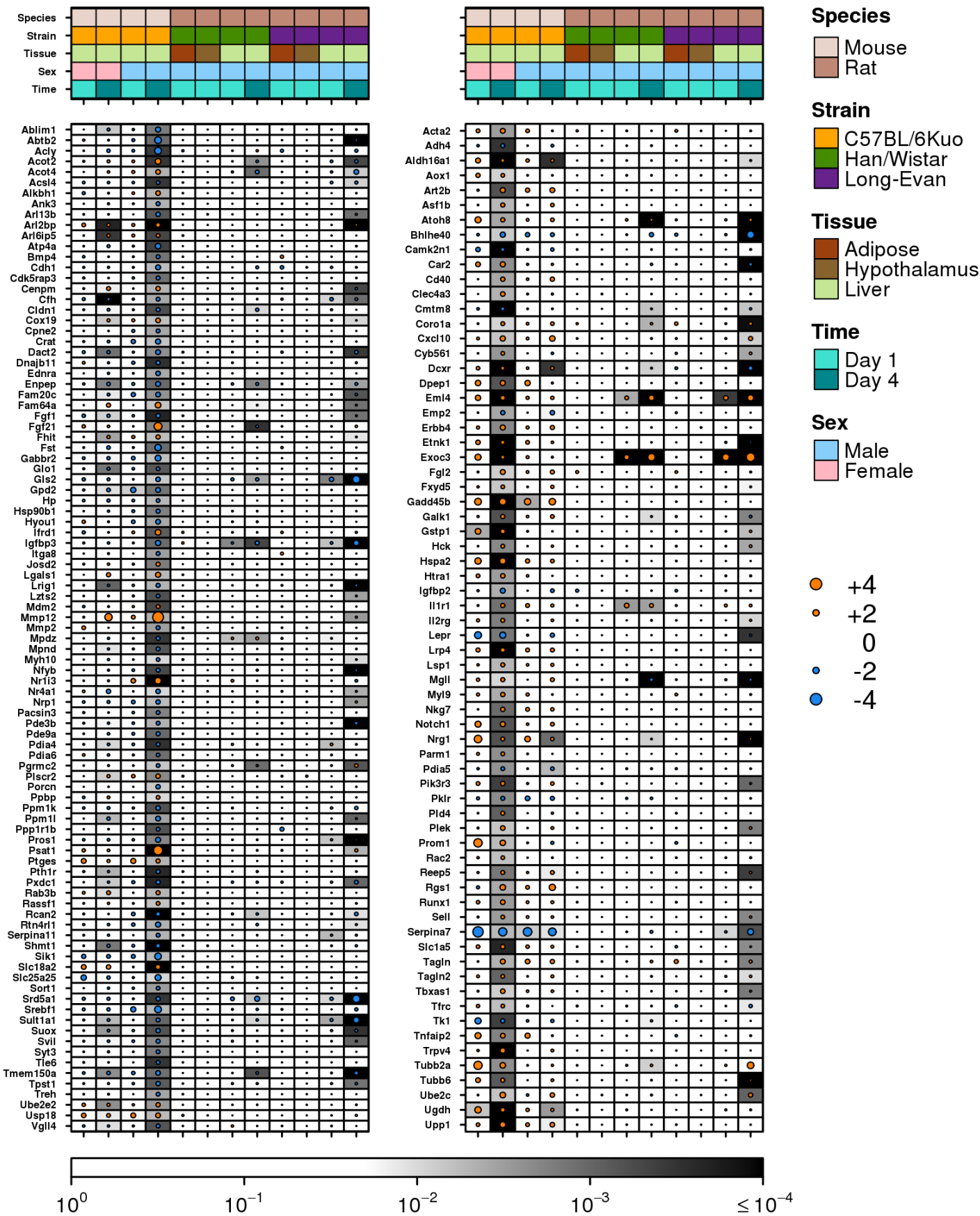

Supplement: Additional file 5: — TCDD-Responsive Genes in Male or Female Mice Examined in Multiple Biological Contexts. Datasets from multiple TCDD toxicity studies in rodents were integrated together to visualize transcriptomic alterations of TCDD-responsive genes (log2|fold-change| > 1 and p adj < 0.01) identified as altered in only (left) male or (right) female livers, 4 days post-exposure to 500 μg/kg TCDD. (PDF 425 kb) [file 12864_2015_1840_MOESM5_ESM.pdf]

# Data Quality Assessment

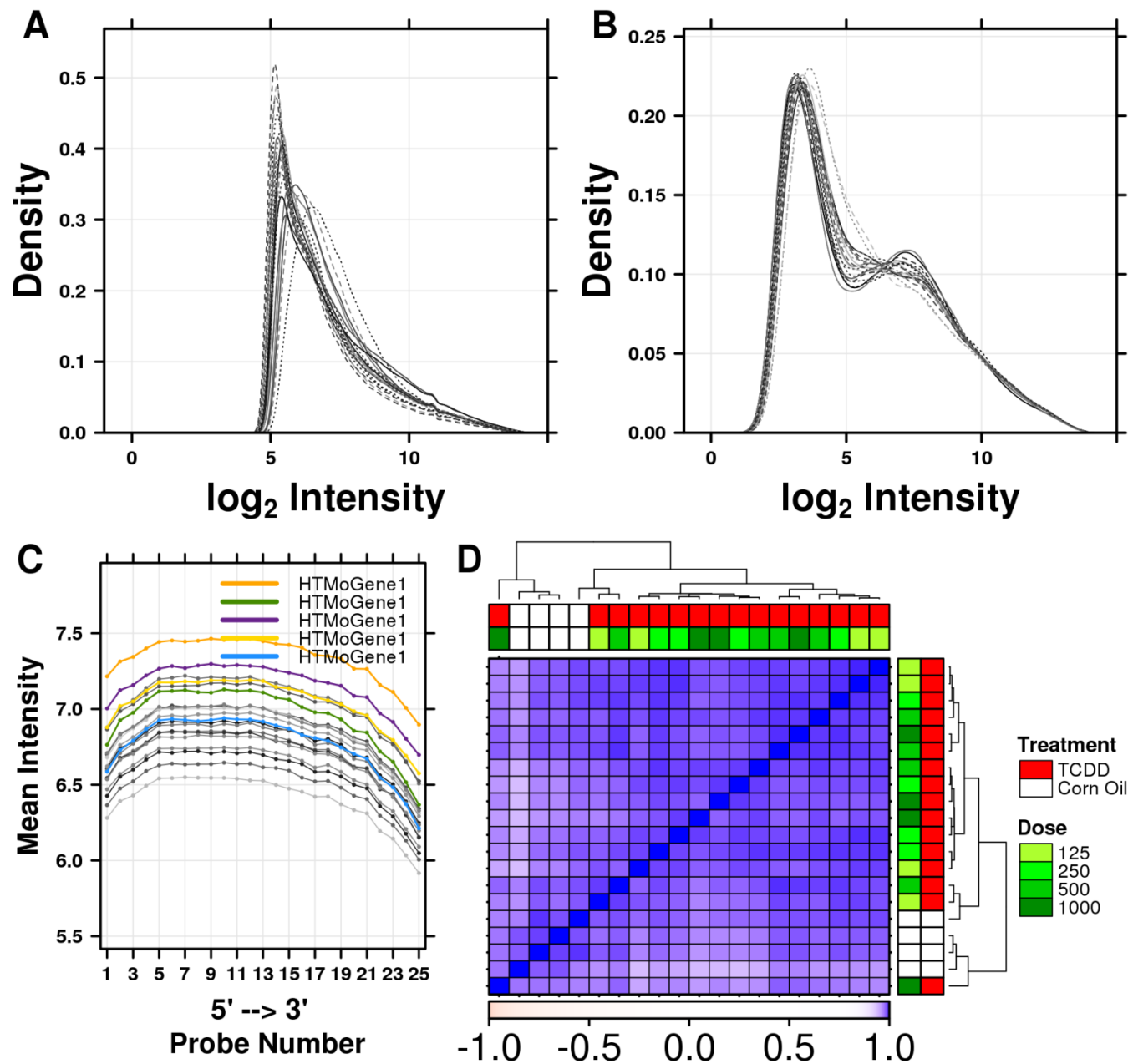

Supplement: Additional file 7: — Array QA/QC (Male Cohort). To verify data quality, the distributional homogeneity of arrays (A) pre- and (B) post-RMA processing was assessed. In addition, (C) RNA degradation was evaluated across probes for each array and (D) the inter-array correlation was examined to identify potential outliers; all arrays appeared highly similar and none were excluded from downstream analyses. (PDF 418 kb) [file 12864_2015_1840_MOESM7_ESM.pdf]

# Data Quality Assessment

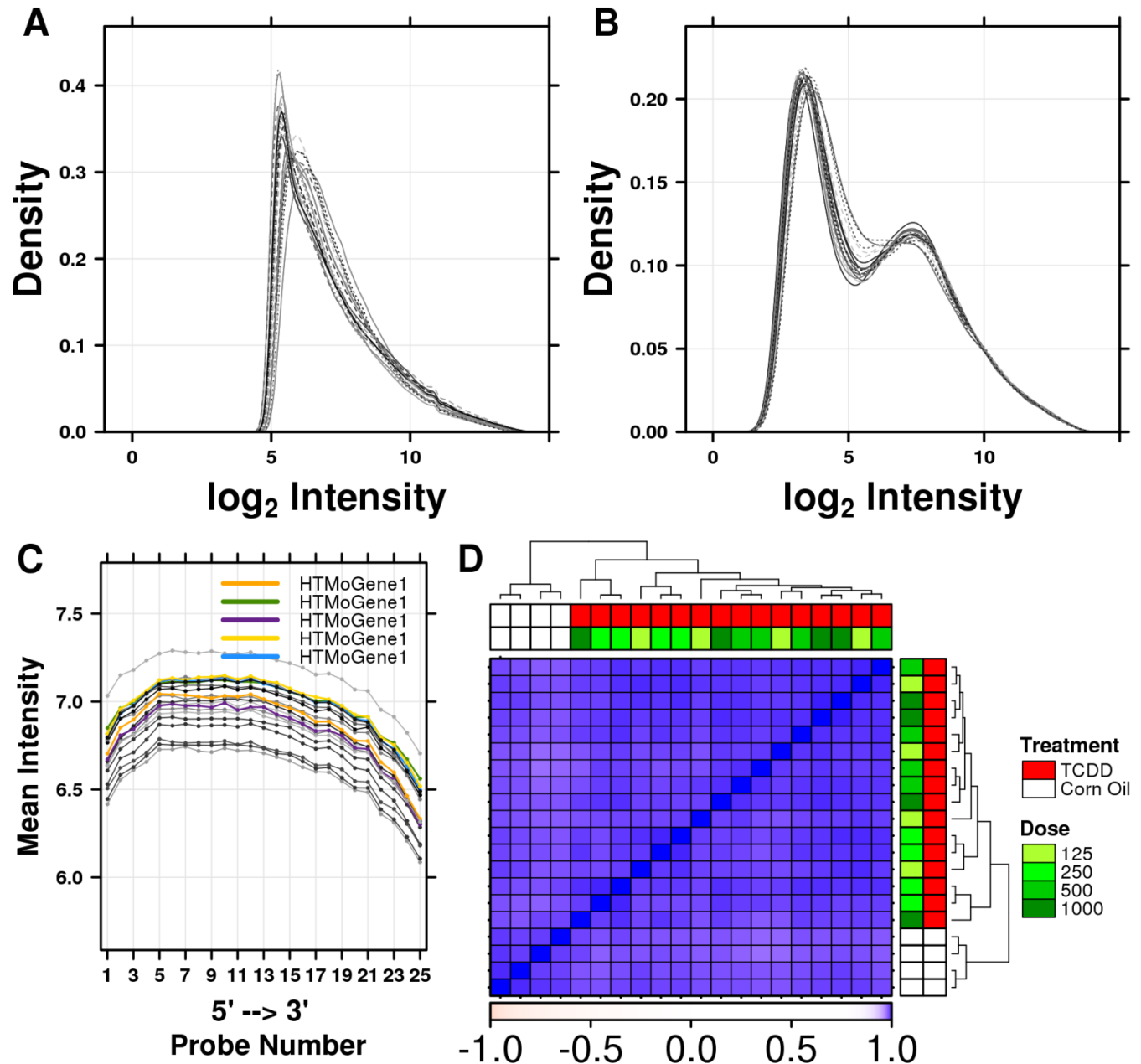

Supplement: Additional file 8: — Array QA/QC (Female Cohort). To verify data quality, the distributional homogeneity of arrays (A) pre- and (B) post- RMA processing was assessed. In addition, (C) RNA degradation was evaluated across probes for each array and (D) the inter-array correlation was examined to identify potential outliers; all arrays appeared highly similar and none were excluded from downstream analyses. (PDF 397 kb) [file 12864_2015_1840_MOESM8_ESM.pdf]
